# Tracking the Effect of Therapy With Single-Trial Based Classification After Stroke

**DOI:** 10.3389/fnsys.2022.840922

**Published:** 2022-05-04

**Authors:** Alessandro Scaglione, Emilia Conti, Anna Letizia Allegra Mascaro, Francesco Saverio Pavone

**Affiliations:** ^1^Department of Physics and Astronomy, University of Florence, Florence, Italy; ^2^European Laboratory for Non-Linear Spectroscopy, University of Florence, Florence, Italy; ^3^Neuroscience Institute, National Research Council, Pisa, Italy; ^4^National Institute of Optics, National Research Council, Florence, Italy

**Keywords:** stroke, wide-field calcium imaging, cerebral cortex, mice, GCaMP6f, single-trial classification

## Abstract

Stroke is a debilitating disease that leads, in the 50% of cases, to permanent motor or cognitive impairments. The effectiveness of therapies that promote recovery after stroke depends on indicators of the disease state that can measure the degree of recovery or predict treatment response or both. Here, we propose to use single-trial classification of task dependent neural activity to assess the disease state and track recovery after stroke. We tested this idea on calcium imaging data of the dorsal cortex of healthy, spontaneously recovered and rehabilitated mice while performing a forelimb retraction task. Results show that, at a single-trial level for the three experimental groups, neural activation during the reward pull can be detected with high accuracy with respect to the background activity in all cortical areas of the field of view and this activation is quite stable across trials and subjects of the same group. Moreover, single-trial responses during the reward pull can be used to discriminate between healthy and stroke subjects with areas closer to the injury site displaying higher discrimination capability than areas closer to this site. Finally, a classifier built to discriminate between controls and stroke at the single-trial level can be used to generate an index of the disease state, the therapeutic score, which is validated on the group of rehabilitated mice. In conclusion, task-related neural activity can be used as an indicator of disease state and track recovery without selecting a peculiar feature of the neural responses. This novel method can be used in both the development and assessment of different therapeutic strategies.

## Introduction

Ischemic stroke is the main cause of severe disability in the elderly population worldwide (Dimyan and Cohen, 2011; Mozaffarian et al., 2015). A recent study shows that the absolute burden of stroke is increasing over the next 30 years in most European countries and therefore people living with some kind of disability are rising too (Wafa et al., 2020). When the lesion involves motor-associated areas, it leads to persistent motor deficits that severely affect patients’ quality of life (Carmichael et al., 2017). After brain injury, the spared circuitry attempts to supply impaired functions by remodeling neural networks (Murphy and Corbett, 2009; Dancause and Nudo, 2011; Zeiler and Krakauer, 2013). These spontaneous mechanisms of recovery have been associated with brain plasticity, which is the ability of the brain to compensate for the loss of function through the reorganization of the neural network (Dancause et al., 2005).

In order to promote post-stroke spontaneous recovery, multiple strategies have been developed such as manipulation of neuronal activity and motor training (Zeiler and Krakauer, 2013; Spalletti et al., 2017; Conti et al., 2021b). However, both the development of new therapies or the selection of the most appropriate treatment for the single patient after stroke rest on the availability of indicators of the disease state that can measure the degree of recovery or predict treatment response or both (Boyd et al., 2017). Among all indicators, indexes that are based on functional measures of neuronal activity have the potential to predict functional recovery within subjects in the same therapeutic group (Nicolo et al., 2015; Wu et al., 2015; Espenhahn et al., 2020) with task related measures being better predictors than the ones based on resting state (Vinehout et al., 2021). Due to these considerations, there is considerable interest in developing systems that can predict functional outcomes based on motor-related neuronal activity. In this framework, preclinical research offers a fundamental tool to test different systems of neuronal activity in order to detect the principal features of neuronal activity distinctive of functional recovery.

Here, we test the idea of employing a single-trial based classification system to provide a measure of both the disease state and track recovery after stroke. To achieve this, we applied this method on calcium imaging data collected from healthy, spontaneously recovering and rehabilitated mice while performing a forelimb retraction task (Allegra Mascaro et al., 2019) in the subacute and chronic stage after stroke. The results from the analysis show that motor-related neural activity can be discriminated with high accuracy in all experimental groups. Moreover, our classifier is capable of distinguishing spatio-temporal features of cortical activity distinctive of a healthy or a stroke subject supporting the idea that this method can provide a measure of the disease state. Finally, to assert if the classifier is able to track recovery after stroke, we used this method to generate an index, the *therapeutic score*, and we validated it against calcium imaging data from a group of mice that underwent rehabilitative treatment that leads to functional recovery. The results of this last analysis show that the therapeutic score is able to separate the rehabilitated subjects from stroke or the control ones since the first week of treatment.

In conclusion, we think that the classification approach we validated in this work has a strong impact on translational research for patients’ stratification and functional evaluation. We believe that this aspect is of great interest in clinics allowing an ongoing modulation of the therapy exploiting an unbiased method thus enhancing functional recovery.

## Materials and Methods

### Mice

**FIGURE 1:**
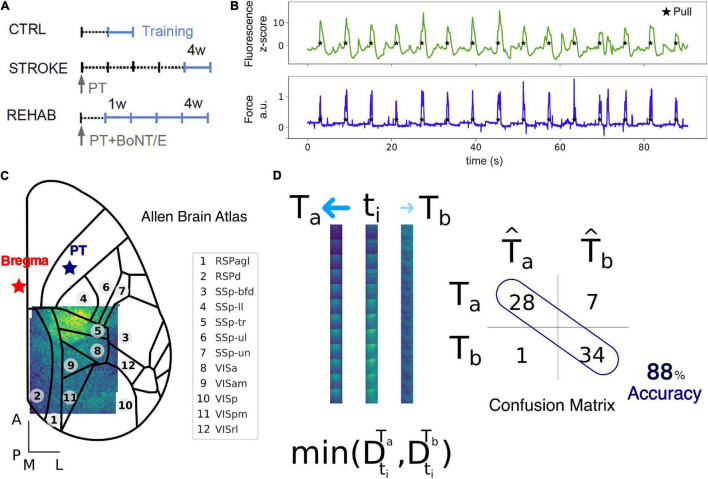
Data collection and Nearest Centroid Euclidean Classifier (NCEC). **(A)** Experimental timeline for the Ctrl, Stroke, and Rehab groups. Light blue lines refer to training weeks. W, week; PT, photothrombosis; BoNT/E, botulinum neurotoxin E. **(B)** Example of fluorescence (green) and force (blue) traces simultaneously recorded during motor training on the M-Platform. **(C)** Field of view with overlaid cortical parcels according to the Allen Brain Atlas. The red star refers to bregma while the blue star refers to the site of photothrombosis. Scale bars in the bottom left corner are 1 mm in length. See Supplementary Table 1 for a list of the region acronym names. **(D)** Schematic representation of the NCEC. For the time window of interest, a single trial, t_*i*_, represented as a sequence of 12 frames, is assigned to the class template T_*a*_ based on the minimum Euclidean distance between the trial and the template. Templates are generated as the average of all other trials for conditions a or b. The results of the classification for each trial are represented by the confusion matrix from which the accuracy can be calculated as the number of correctly classified trials over the total number of trials.

All experimental procedures were performed in accordance with directive 2010/63/EU on the protection of animals used for scientific purposes and approved by the Italian Minister of Health, authorization n.183/2016-PR. Mice were housed in clear plastic cages under a 12 h light/dark cycle and were given *ad libitum* access to water and food. In this study, a transgenic mouse line [C57BL/6J-Tg(Thy1GCaMP6f)GP5.17Dkim/J] expressing a genetically encoded fluorescent calcium indicator under the control of the Thy-1 promoter was used. Mice were identified by earmarks and numbered accordingly. Animals were randomly assigned to three experimental groups: Control (4), Stroke (6), and Rehab (6) (Figure 1A). Calcium imaging data were recorded for our previous work (Allegra Mascaro et al., 2019). Each group contained comparable numbers of male and female mice (weighing approximately 25 g). The age of mice was consistent between the groups (ranging from 4 to 8 months).

### Surgical Procedures

All surgical procedures were performed under Isoflurane anesthesia (3% induction, 1.5% maintenance, in 1.5 L/min oxygen). The animals were placed into a stereotaxic apparatus (Stoelting, Wheat Lane, Wood Dale, IL, United States) and, after removing the skin over the skull and the periosteum, the primary motor cortex (M1) was identified (stereotaxic coordinates +1.75 lateral, +0.5 anterior to bregma). Five minutes after intraperitoneal injection of Rose Bengal [0.2 mL, 10 mg/mL solution in Phosphate Buffer Saline (PBS); Sigma Aldrich, St. Louis, MO, United States], the targeted region of the cortex (M1) was illuminated through the intact skull for 15 min with a white light from an LED lamp (CL 6000 LED, Carl Zeiss Microscopy, Oberkochen, Germany) linked to a 20× objective (EC Plan Neofluar NA 0.5, Carl Zeiss Microscopy, Oberkochen, Germany) to induce unilateral stroke in the right hemisphere (Conti et al., 2021a). Control mice were injected with 0.2 mL of saline and then illuminated as the others. We choose a photothrombotic stroke model as a non-invasive technique to induce a targeted ischemic stroke highly reproducible. Thirty days after photothrombosis, a subgroup of animals was perfused first with 20–30 mL of 0.01 M PBS (pH 7.6) and then with 150 mL of paraformaldehyde 4% (PFA, Aldrich, St. Louis, MO, United States). The fixed brains were then cut using a vibrating-blade microtome (Leica, Germany) to obtain 100 μm thick coronal sections that were used for immunostaining of NeuN (1:200, Millipore, Germany). Lesion volume located in the primary motor cortex of the right hemisphere was comparable between animals (1.2 ± 0.1 mm^3^, average ± SEM). Botulinum neurotoxin E (BoNT/E) injections in rehab mice were performed during the same surgical session of the photothrombotic lesions. We used a dental drill to create a small craniotomy over M1 of the healthy hemisphere (ML: −1.75; RC: +0.5). Then 500 nL of BoNT/E were delivered in two separate injections. Cover glass and an aluminum head-post were attached to the intact skull using transparent dental cement (Super Bond, C&S). Afterward, the animals were placed in their cages until full recovery.

### Motor Training Protocol on the M-Platform

Each mouse was allowed to become accustomed to the apparatus before the beginning of the motor training on the M-Platform. The M-Platform is a robotic system that encourages mice to perform a retraction movement of their left forelimb and has been described before (Spalletti et al., 2014). Here we give a brief description of the paradigm. The task consisted of up to 15 cycles of passive extension of the affected forelimb followed by its active retraction triggered by an acoustic cue. A liquid reward (milk) was then delivered 0.5 s after each complete pull to motivate mice during the training session. Control mice performed 1 week (five sessions) of motor training, Stroke mice performed 1 week (five sessions) of daily training starting 26 days after injury. Rehab mice performed 4 weeks (20 sessions) of daily sessions starting 5 days after photothrombosis. Force and position data were collected in each session through a NI-6251 USB DAQ card (National Instruments, Austin, TX, United States) and sampled at 100 Hz.

The M-Platform was designed to allow mice in all conditions (before the stroke, right after the stroke, and during the weeks under all treatments) to easily perform the motor task from the very first session by applying similar forces. For the same reason, this robotic device is not suitable to evaluate post-stroke functional impairment.

### Wide-Field Fluorescence Microscopy

The highly versatile custom-made wide-field imaging setup (Crocini et al., 2016; Turrini et al., 2017; Conti et al., 2019) was equipped with a 505 nm LED (M505L3 Thorlabs, Newton, NJ, United States) deflected by a dichroic filter (DC FF 495-DI02 Semrock, Rochester, NY, United States) on the objective (2.5× EC Plan Neofluar, NA 0.085, Carl Zeiss Microscopy, Oberkochen, Germany). The fluorescence signal, selected by a bandpass filter (525/50 Semrock, Rochester, NY, United States), was collected on the sensor of a high-speed complementary metal-oxide semiconductor (CMOS) camera (Orca Flash 4.0 v 2.0 Hamamatsu Photonics, NJ, United States). Images (512 × 512 pixels, pixel size 9 μm for a field of view of 4.6 mm × 4.6 mm) were acquired at 25 Hz. At the beginning of each session, the camera was triggered by a TTL pulse generated by the NI-6251 card of M-Platform to allow the synchronization of the calcium and the force data.

### Data Processing and Analysis

All data preprocessing and analysis were implemented in Python (Python Software Foundation) using the numpy, scipy, and sklearn packages.

#### Preprocessing

**FIGURE 2:**
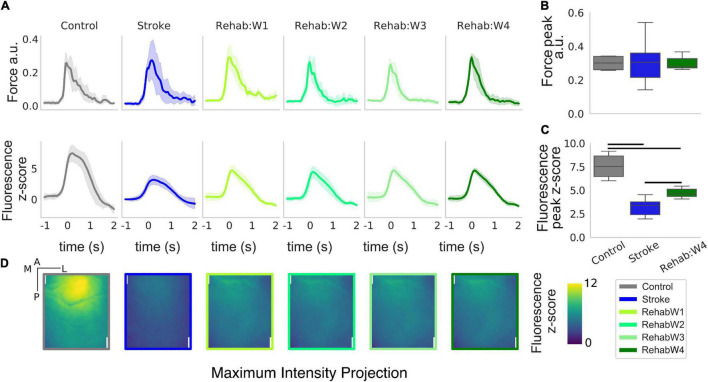
The peak and the spatial distribution of the calcium transient are partially recovered in the rehabilitation group. **(A)** Force and calcium profiles, top and bottom rows, respectively, in response to the rewarded pull for all treatment groups. Time is referenced to the end of the retraction phase which precedes the force peak. Boxplot of the peak force **(B)** and peak fluorescence **(C)** for all subjects. For each box, the middle line represents the median. The box spans the interquartile range (IQR) (25–75%) while the whiskers extend to show the rest of the distribution excluding points that are far apart 1.5 times the IQR. Horizontal bars denote statistically significant differences among groups. **(D)** Maximum intensity projection, the maximum intensity of the fluorescent stack in the time window ranging from –1 to 2 s after the rewarded pull computed for each individual pixel. Scale bars in the top right corner of the first figure are 1 mm in length.

Data acquired during each recording session for each mouse (Figure 1A) was processed offline using custom routines. Each such dataset consisted of up to 15 cycles of active retraction movements on a slide triggered by the emission of an acoustic cue. To ensure the consistency of the field of view across sessions and across mice, each frame of the fluorescence data was first downsampled with a factor of 9 resulting in a pixel size of 63 μm and then offline registered by aligning each frame to two reference points (corresponding to bregma and lambda) that were previously marked on the dental cement during the surgery procedure. After the image registration across all days, subjects and groups, we kept only the regions in the field of view that were common to all sessions (Figure 1C). The more marginal regions were excluded from the analysis and thus the final area analyzed contains at most a very small portion of the lesion. To be able to compare changes in fluorescence across subjects and groups we performed a *z*-score normalization of the raw fluorescence series on a for each individual pixel. The *z*-score fluorescence series were then epoched, ±3 s, around the rewarded pull (Figure 1B), which corresponds to the end of the retraction phase and precedes the peak force exerted by the animal (Figures 1B, 2A top row). Trials where the animals exerted force in the window −2 to −0.5 before the rewarded pull were discarded to ensure a rest condition before the rewarded pull without spurious calcium activity. The total number of trials per group used for the analysis were: 130 (Control), 181 (Stroke), 167 (Rehab:W1), 193 (Rehab:W2), 164 (Rehab:W3), and 189 (Rehab:W4).

#### Nearest Centroid Euclidean Classifier

Single-trial classification was performed using a supervised Nearest Centroid Euclidean Classifier (NCEC). We selected this family of classifiers because they are simple to implement and understand, are commonly employed as benchmark classifiers and used when the number of features are much bigger than the number of samples (Hastie et al., 2009). In such classifiers, classification of a single trial from the test set, t_*i*_, is performed by measuring the Euclidean distance between the trial and the classes templates and assigning the trial to the class with the minimum distance. Templates are constructed by taking the average of a set of trials, the training set, for classes a or b (Figure 1D). The way in which the train and the test set are chosen lead to different classification validation schemes. Here we use two different schemes: (1) *leave-one-trial-out* (loto), where each trial is tested using templates generated by all other trials in the two conditions and (2) *leave-one-subject-out* (loso), where the trials of one subject are tested using templates generated by trials of all other subjects. The results of the classification for each trial are represented by the confusion matrix from which the accuracy can be calculated as the number of correctly classified test trials over the total number of test trials. Therefore, for the leave-one-trial-out scheme the accuracy is the total number of trials classified correctly over the total number of trials. Instead, for the leave-one-subject-out scheme the accuracy is the average of the accuracy obtained for each subject left out. We employ the leave-one-out-trial to extract all possible information from the dataset and use it to measure the contribution of the different cortical regions. Instead, the leave-one-subject-out scheme was used to test the stability of the discrimination across different groups of treatments.

#### Detection and Discrimination of Rewarded Pull

We performed two separate types of classifications: *pull detection* and *pull discrimination*. For the *pull detection*, we wondered if the calcium transients carry any information about the pull event with respect to the baseline condition. To test this, we performed a binary classification employing both validation schemes (loto and loso) selecting as template for the baseline a fixed sequence of 12 frames starting at −2 s from the rewarded pull, and as a template for the rewarded pull a sliding sequence of 12 frames starting at −1 to 0.5 s after the rewarded pull. Therefore a single trial (t_*i*_ Figure 1D) consisted of a sequence of 12 frames that captured the spatiotemporal evolution of the calcium dynamic in a 1 s window. Performing the classification with a sliding time window for the pull template we are then able to observe the time course of the accuracy and identify the window with the highest accuracy.

For the *pull discrimination* case, we wanted to test if we were able to discriminate if a pull event was performed by a healthy or a stroke subject to assess if the calcium transient around the event carries any information about the group to whom the subject belongs to. To test this we performed a binary classification employing both classification schemes selecting a sequence of 12 frames from the healthy subjects as trials of one template and another sequence of 12 frames from the stroke subjects as trials of the second template. As in the case of the pull detection classification, a single trial consisted of a sequence of 12 frames. We then performed this classification using a sliding time window starting from −2 to 0.5 s after the rewarded pull. It is important to note, that because there are more trials for the stroke group compared to the control group, i.e., 130 vs. 181, respectively, a classifier that ignore any structure in the data and just guesses each time the most numerous class (stroke) will achieve an accuracy of 58%, 181/(130 + 181), which represents the baseline accuracy for this classification problem.

Finally, to test the contribution of the cortical areas in the field of view, for both pull detection and discrimination, we performed a separate classification for each cortical parcel in the field of view plus one for the entire field of view.

#### Evaluation of the Effect of Therapy Using the Nearest Centroid Euclidean Classifier

We employed the classifier built for the pull discrimination and classified single trials generated by the mice in the rehabilitative paradigm to see if such classifier can assess the effect of therapy based only on the calcium transients. In particular, first we selected the classifier that yielded the maximum accuracy when discriminating between healthy and stroke subjects. Then we used this classifier to compute a therapeutic score that corresponds to the number of trials that are classified as healthy over the total number of trials generated by the subject. In this way each subject tested could have a score that ranged from 0 to 1 with 1 meaning that all trials produced by the subject are classified as healthy controls. It is important to note that the classifier training set is generated only from trials of the control or stroke subjects and therefore if a trial is classified as healthy it means that the trial is closer to the healthy centroid than the stroke one. The therapeutic score obtained for the Rehab group was compared to the same score obtained when performing a *loso classification* for each subject of the other two groups. Finally, to test the contribution of the different cortical areas in the field of view, we performed a separate classification for each cortical parcel in the field of view plus one for the entire field of view.

#### Statistical Analysis

All analyses were performed in Python using the scipy and statsmodels packages. For the analysis of the peak force and peak fluorescence triggered by the rewarded pull a one-way analysis of variance (ANOVA) was performed with the main factor “Group” with levels “Control,” “Stroke,” and “Rehab:W4.” When considering the time course of the rehabilitative therapy, a one-way repeated measure analysis of variance (ANOVA-RM) was performed with the main factor “Week” with levels “Rehab:W1,” “Rehab:W2,” “Rehab:W3,” and “Rehab:W4.”

For the analysis of the peak accuracy in the pull detection discrimination a one-way ANOVA was performed with the main factor “Group,” while a one-way ANOVA-RM was performed on the longitudinal data of the rehabilitation group with main factor “Week.”

For the analysis of the recovery score a one-way ANOVA was performed with the main factor “Group,” while a one-way ANOVA-RM was performed on the longitudinal data of the rehabilitation group with main factor “Week.” For all ANOVAs, *post hoc* analyses were performed, only when the main effect was statistically significant, for all pairwise comparisons among marginal means of the respective factors. The *p*-values were corrected with the Holm–Sidak method to account for the family wise error rate.

Results were considered statistically significant if the corresponding *p*-value was less or equal to 0.05.

## Results

### The Rehabilitative Paradigm Recovers Some Features of the Response Compared to Healthy Subjects

We extracted force and calcium transients in response to the retraction of the left forelimb triggered by the acoustic cue for all groups of subjects considered (Figure 2). While the peak of the force profiles shows no changes across the three groups (Figure 2B) [one-way ANOVA *F*(2,13) = 0.016, *p* = 0.98], changes in the calcium transient are clearly evident (Figure 2C) [one-way ANOVA *F*(2,13) = 22.5, *p* < 0.001]. Indeed, stroke greatly reduces the peak of the calcium transients in response to the forelimb retraction compared to healthy subjects (*p* = 0.002). In fact, the peak amplitude in stroke subjects is as high as 42% of the peak in healthy subjects. The effect of the treatment increases the peak response with respect to the stroke condition (*p* = 0.009) but not enough to reach the control level (*p* = 0.005), in fact, the peak amplitude in the rehabilitation group accounts for 62% of the one observed in the healthy subjects. Finally, there were no changes in both the force and the calcium transients for the longitudinal data of the rehab (Supplementary Figure 1).

When considering the regions activated during the retraction phase, healthy subjects display peak activation in the somatosensory areas representing the trunk, upper and lower limb (refer to Figure 1C for the area in the field of view). This peak activation is lost in stroke subjects (Figure 2D, panel 2) resulting in a sparse activation, while the peak activation is only partially regained in rehabilitated subjects (Figure 2B, panel 3–6).

Overall the rehabilitative treatment seems to recover partly of the peak response and some spatial aspects of the spatio-temporal pattern observed in control animals (supporting our idea that such features can be used to evaluate treatments therapies).

### Rewarded Pull Can Be Detected With High Accuracy in All Treatments Groups

**FIGURE 3:**
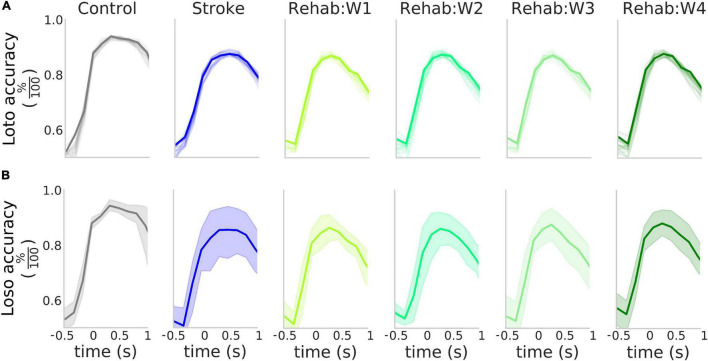
High accuracy in the detection of the rewarded pull. **(A)** Accuracy of the detection of the rewarded pull for the different experimental groups as a function of the time window considered in a loto scheme. Solid lines represent the accuracy obtained when considering the entire field of view, while the shaded lines represent the result of the classification for the 12 parcels in the field of view. **(B)** Accuracy of the detection of the rewarded pull for the different experimental groups as a function of the time window considered in a loso scheme. Solid lines represent the average across all tested subjects while the shaded region represents the 95% confidence interval of the mean. In both **(A,B)** the value of the *x*-axis represents the time of the last frame in the sliding window used for the analysis.

Since the different therapies affected the peak response of the calcium transients (Figure 2A), we wanted to test if such transients, at a single trial level, can be used to detect the retraction movement onset from the baseline activity within each treatment group. For each group, we performed a binary classification with a NCEC to discriminate between baseline activity and retraction phase at the single-trial level (see methods). To identify the time window that yields the best discrimination performance, we performed this classification by changing the response centroid by sliding the response window over a range starting from −1 to 2 s which encapsulated the entire calcium transient (see Figure 2). The results of such analysis are shown in Figure 3A (solid dark lines). As it can be seen the peak performances in terms of the highest accuracy are obtained when selecting a time window with initial time at the onset of the retraction movement. In fact, for all three groups in this window accuracies of about 90% can be achieved. To check how all cortical parcels contributed to the detection, we repeated the same analysis for all the areas in our field of view for each area independently. The results of such analysis are shown in Figure 2A by the lighter lines in each panel denoting that for the purposes of detection each area contributed almost in the same way for all groups.

We validated this classification paradigm against a leave-one-subject out scheme. This was obtained by excluding from the training set all trials from one of the subjects and repeating the classification process. The results of this analysis (Figure 3 solid bold lines) show that on average as high as 85% accuracy can be achieved in all three experimental groups and the peak detection accuracy is not influenced by the experimental group (Supplementary Figure 2). The Control group displayed less variability in the accuracy with respect to both the Stroke and the Rehab group (possibly due to variability in the lesion or individual response to the treatment). However, despite this variability, the high degree of accuracy obtained by the classifiers suggests that the calcium transients carry information about the retraction phase with accuracy that peaks at the movement onset.

### Single-Trial Calcium Activity in Response to the Rewarded Pull Can Discriminate Between Healthy and Stroke Subjects

**FIGURE 4:**
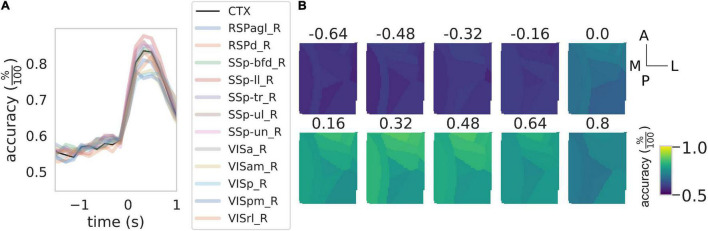
Rewarded pull can be used to discriminate between healthy control and stroke subjects. **(A)** Time course of the accuracy of the discrimination between control and stroke subjects as a function of the time window considered. Black solid line represents the result of the discrimination over the entire field of view while the shaded colored lines represent the result for the same classification for the 12 cortical parcels in the field of view. See Figure 3 for the definition of the *x*-axis. **(B)** Time course of the accuracy as a function of the time window for the 12 areas in the field of view. Scale bars at the far right are 1 mm in length.

We wondered if in addition to the movement onset calcium transients carry information about the treatment group. Indeed as seen in Figure 2, different groups have different calcium transients, however, this does not imply that at the single-trial level one can discriminate between groups nor can predict the accuracy of such a prediction from the average traces shown before. To this aim, we performed a binary classification with another NCEC with class centroids made with the responses around the retraction phase in healthy and stroke subjects. To find the optimal discrimination time window we performed this classification by rolling both response windows, one for the healthy and one for the stroke, from −1.5 to 2 s after movement onset. The results of this analysis (Figure 4A) show that peak accuracy as high as 82% can be achieved during the retraction phase while before movement onset, the classification accuracy is almost 58%, which is not different from the baseline accuracy expected for this classification problem. In addition, different regions seem to contribute differently to the discrimination (Figures 4A,B). In particular, areas nearest to the injury site have discrimination performances as low as 74% while areas away from the injury have accuracy as high as 88%.

Overall these results indicate that calcium transients can be used to discriminate between healthy and stroke subjects and that almost the total accuracy of the discrimination performances are due to calcium transients during the retraction phase.

### Single-Trial Classification Can Be Used to Assess Recovery in a Group of Rehabilitated Subject

**FIGURE 5:**
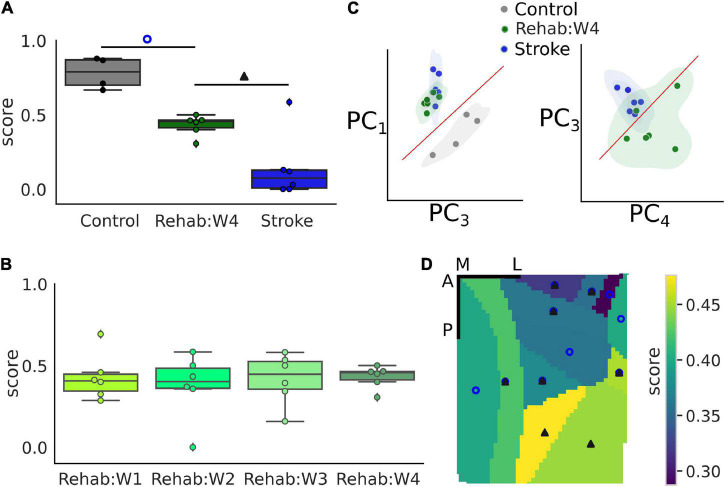
Single-trial classification can discriminate between treatment groups. **(A)** Boxplot of the recovery score for the different experimental groups. Refer to Figure 2 for a detailed description of the boxplot. The empty blue circle denotes statistically significant differences between the control and the Rehab:W4 group while the solid triangle denotes statistically significant differences between the Rehab:W4 and the Stroke group. Single dots represent the score for the single subject. **(B)** Boxplot of the recovery score across weeks for the rehabilitation group. Single dots represent the score for the single subject. **(C)** Representation of the trials in the principal component space. Each dot represents the centroid of all trials for a single subject. **(D)** Map of the average score of the rehabilitation group for the 12 different parcels. Empty circles and filled triangles denote statistically significant differences as indicated in A. Scale bars in the top right corner are 1 mm in length. For both **(A,D)** the control group is always statistically significantly different from the stroke group (see Supplementary Table 2 for a list of the areas and the corresponding *p*-values).

Finally, we wanted to test if the NCEC can be used to evaluate the effectiveness of therapeutic interventions. As a proof of concept, we used the classifier trained on healthy and stroke subjects and validated it against the individual subjects of the Rehab group. In particular, each tested subject was assigned a score from 0 to 1 which represents the percentage of trials classified as healthy by the NCEC. These scores were compared against the ones obtained when performing the same with a loso scheme for control and stroke subjects. Figure 5A shows the results of this analysis when considering the calcium transient signal on the entire field of view. The score was strongly influenced by the group [one-way ANOVA *F*(2,13) = 31.73, *p* < 0.001]. In fact, subjects in the rehabilitation group have a score higher than the subjects in the stroke group (*p* = 0.011) but lower than that of the Control group (*p* < 0.001). On the contrary, when looking at the time course of the score across the different weeks for the Rehab group (Figure 5B), the score was similar across weeks for all subjects of the group [one-way ANOVA-RM, *F*(3,15) = 0.0735, *p* = 0.973].

To gain some insights into the classification process that produces the score, we used the principal component analysis (PCA) to reduce the dimensionality of the data into the first four components, which accounted for 84% of the total variance observed. Figure 5C shows two projections of the data where each dot corresponds to the centroid of the trials of each subject. These projections were selected by hand, among the 6 possible ones, as those projections that showed a high degree of separation between the experimental groups. From these projections, it can be seen that subjects of the Rehab groups tend to form a set that lies between the control and stroke sets although closer to the stroke than to the control group as can be seen clearly in the second graph of Figure 5C which shows some overlap between the two sets.

Lastly, we wondered if different regions of the field of view contributed differently to the generation of the score (Figure 5D). In particular, statistically significant differences among all groups are present in 5 of the 12 areas in the field of view (RSPagl, SSp-ll, SSp-tr, SSp-ul, and VISrl), 5 areas displayed statistically significant differences between the control and Rehab groups (RSPd, SSp-bfd, SSp-un, VISa, and VISam), and 2 areas displayed statistically significant differences between the Rehab and the Stroke group (VISp and VISpm) (see Supplementary Table 2 for the corresponding *p*-values). Overall somatosensory areas seem to achieve better discrimination capability than visual and associative areas. The discrimination map seems to be due mostly to the value of the score for the Rehab group, when the score is low there is no significant difference between this group and the stroke one. Conversely, a high score increases the *p*-value for the difference between the Rehab and the Control group. It is interesting to note that the further from the injury site the higher is the score for the Rehab group.

In conclusion, calcium transients can be used to assess the effect of treatment following the induction of a focal cortical stroke with areas that are distal to the injury site contributing more to the value of the score.

## Discussion

In this study, we apply single-trial classification methods to wide-field calcium imaging collected from the dorsal cortex of healthy, stroked, and rehabilitated mice while performing a forelimb retraction task. First, we show that the neural activation during the reward pull can be detected with high accuracy with respect to the background activity in all experimental groups. We then show that the single-trial responses to the reward pull can be used to discriminate between healthy and stroke subjects. Also as opposed to the detection case where all areas contributed equally to the detection, areas closer to the injury site displayed higher discrimination capability compared to areas that are far from the stroke induction site. Finally, we show that the classifier built to discriminate between controls and stroke can be used to assess recovery in a group of rehabilitated subjects.

### Methodological Considerations

Nearest Centroid Classifiers represent a family of classifiers that are simple to implement, understand, and are commonly employed as benchmark classifiers (Levner, 2005; Hastie et al., 2009). They are mostly used when the number of features is much bigger than the number of samples (Tibshirani et al., 2002) such as for the dataset in our study. For these reasons we believe that the values of accuracy reached in this work represent a lower bound and that higher values can be achieved by using more sophisticated classification approaches, maybe combining them with feature reduction techniques to reduce the dimensionality of the data.

The main advantages of Nearest Centroid Classifiers are low computational cost both during the training and the testing phase, which makes them extremely useful for datasets with a high number of features (Levner, 2005). However, such an advantage comes at the cost of not being able to identify, as a direct result of the classification, the features that are strictly related to the performance of the classification, in other words, we only know that the information contained in the data it is sufficient for the discrimination. To overcome this problem it is possible to use surrogate data (Lancaster et al., 2018; Allegra Mascaro et al., 2020), in particular, it is possible to generate a dataset with the same distribution of the original data but that lacks the property of interest and use this data for the discrimination.

Single-trial classification techniques lay at the heart of brain–machine interfaces (BMI) systems that have been largely employed in neurorehabilitation after stroke (Buch et al., 2008; Broetz et al., 2010; Ramos-Murguialday et al., 2013). In such systems, Classification techniques are used to provide neurofeedback to the subject and this is sufficient to improve the outcome of stroke patients as proved at both the research and the clinical trial level. As a whole, BMI systems are used to measure functional recovery and neural plastic changes (Venkatakrishnan et al., 2014). Here we show that by using single-trial classification alone it is possible to measure the therapeutic effect of the rehabilitative treatment. In other words, the evolution in neural dynamics due to plasticity after stroke can be used not only to provide neurofeedback but also to quantitatively assess the effect of therapeutic interventions.

### Detection and Discrimination of the Rewarded Pull Across Different Treatments

In our study, we obtain high detection accuracy for the reward pull compared to the baseline when performing a loso classification scheme for the three experimental groups. In addition, our investigation reveals that after an ischemic insult there is an increase in variability, both in the Stroke and Rehab groups compared to the Control group. This result suggests the loss of a coherent functional activation of cortical circuitries during the execution of the pulling task as a consequence of structural disruption induced by the damage. In fact, as previously observed both in animal models (Harrison et al., 2013; Allegra Mascaro et al., 2019; Conti et al., 2021b) and in humans (Siegel et al., 2016), after stroke a loss of modularity takes place resulting in a widespread activation. In particular, as we demonstrated in previous studies (Allegra Mascaro et al., 2019; Conti et al., 2021b), in absence of rehabilitative intervention the motor representation covers almost the entire ipsilesional cortex of untreated mice (Stroke group) while combined rehabilitation promotes the refocusing of motor control. The loss of modularity induced by the damage might be a possible explanation of the variability observed in the Stroke and Rehab groups that is only partially recovered during rehabilitation.

When performing the detection of the reward pull, our results suggest that all cortical parcels in the field of view contributed equally to the accuracy of the detection across all experimental groups, meaning that all parcels participated in the response to the reward pull and that this response is different from the baseline condition. This is consistent with other studies that report widespread neuronal activity over the cortical mantle of animals engaged in behavioral tasks (Allen et al., 2017; Steinmetz et al., 2019; Quarta et al., 2021). In particular, this is consistent with our previous findings where we observed a large wave traveling across most of the field of view especially during the reward pull (Cecchini et al., 2021). Therefore the high degree of accuracy obtained for the detection of the pull for each parcel could be the expressions of traveling waves propagating over the cortex independently of the experimental group.

When using the calcium transient in response to the reward pull to classify the group (healthy vs. stroke), no areas have a discrimination accuracy near to chance meaning that all parcels in the field of view have peculiar calcium transients that are different between the two groups. This is well in agreement with other data from both humans and animals models that demonstrate that the ischemic event alters the neural dynamics in the perilesional areas as well as the interconnected areas over the entire cortex (Dijkhuizen et al., 2001; Murase et al., 2004; Ovadia-Caro et al., 2013). However, not all areas contributed equally to the discrimination between the healthy and stroke subjects. In fact, the areas near the injury site together with the retrosplenial regions show a high discrimination accuracy while primary and association visual areas have low accuracy values. Indeed most of the plastic changes after stroke take place in the perilesional area where they manifest mainly as alterations in the excitatory/inhibitory balance together with the remapping of functional areas (Carmichael et al., 2005; Harrison et al., 2013; Allegra Mascaro et al., 2019). With this in mind, it is reasonable to expect that areas that are closer to the injury would display a more altered activity with respect to areas away from the injury site hence contributing more to the accuracy of the discrimination.

The high accuracy values obtained for both the detection and the discrimination of the reward pull implies that the neural activity of a single trial is similar to the average of all other trials suggesting a rather stable neural representation of the reward pull across days and subjects of the same group. At first, this might seem counterintuitive, especially because of the high variability that is usually found in the activity of neural systems in response to the same stimulus (Adrian and Zotterman, 1926; Shadlen and Newsome, 1998; Vogel et al., 2005; Scaglione et al., 2011). However, recent studies in both mice and primates support the alternative hypothesis of a stable representation of neural responses in motor cortices that last for days or weeks with motor learning contributing to the stabilization of the activity (Carmena et al., 2005; Chestek et al., 2007; Ganguly and Carmena, 2009). In this sense, the motor training used in this study may have contributed to the stabilization and therefore to the high accuracy values observed. Neural representations are found to be more variable in sensory cortices, in both visual and sensory cortices (Mank et al., 2008; Margolis et al., 2012) the activity of single neurons can be highly heterogeneous, however, when the responses of these neurons are grouped into a population response such population displays a rather stable neural response (Margolis et al., 2012). Therefore even if at the single neuron level the neural representation of the reward pull is not stable the wide-field calcium dynamic which presumably represents the aggregate activity of hundreds if not thousands of neurons in one pixel of the field of view could result in a stable activity over time.

### Single-Trial Classification to Assess the Therapeutic Effect of Treatments

The single-trial classification of rewarded pull highlights a strong discrepancy in the assessment of calcium transient and force signals. Indeed, the score produced by the classification is more accurate in discriminating different conditions (i.e., health, untreated, and rehabilitated animals) with neuronal activity with respect to forces. In fact, as observed previously (Conti et al., 2021b), we do not find any substantial change in the force exerted by the animals during the motor training on the robotic device regardless of the experimental group. Indeed, this mechatronic device is conceived to allow a reproducible motor training of the affected forelimb, not to assess motor impairment. Moreover, the training we designed in these experiments was easy to perform, in order to allow all mice to execute the pulling task, regardless of gender, age, and weight. Therefore, mice in all conditions (before a stroke, right after a stroke, and along the weeks under all treatments) can perform the motor task from the very first session by applying similar forces. Future studies where pulling resistance is modulated over the rehabilitation period will be useful in assessing motor performance by means of the robotic platform.

Finally, we validate the capability of NCEC to assess the effectiveness of post-stroke rehabilitative treatments. The single-trial classification shows that the score decreased in untreated animals is partially recovered after 4 weeks of rehabilitation. However, in the rehab group, no differences along the time course were observed. This trend is probably due to the effect of the combination of temporal inactivation of the homotopic contralateral cortex and repetitive motor training. More in detail, we think that the BoNT/E effect confined in the acute phase after stroke has a boosting role in the first week after the insult, as the score trend shows. Moreover, the repetitive motor training performed since the acute phase after the ischemic insult could play a role in the stabilization of the plastic changes and indirectly in the neural responses.

Indeed, this result matches the behavioral outcomes previously observed by Spalletti et al. (2017) through the Gridwalk test. In their study, the error percentage revealed by the behavioral test remains quite stable starting from the first week of rehabilitation. Similarly, the score obtained during the 4 weeks of rehabilitation settles on a value intermediate between Ctrl and Stroke groups as early as the first week after stroke.

The value of the therapeutic score for the Rehab group close to the chance level could be interpreted as the result of a poor classification performance. However, we strongly believe that this is not the case for two different reasons. First, because we observe the existence of a map of the therapeutic score in the field of view, in fact, there is a gradual increase in the value of the score from 30 to 47% the further the area from the injury site. And, furthermore, this is well in agreement with the fact that most of the plastic changes happen in the perilesional region (Harrison et al., 2013; Allegra Mascaro et al., 2019). Second, the results in Figure 5D show that the three experimental groups tend to cluster with the Rehab group being closer to the Stroke group than to the Control group. This implies that there is a good degree of similarity across the subjects of a single group which supports the use of a NCEC and supports the fact that the score must be different across the three groups. Finally, the separation among the three groups supports the idea that functional recovery is not necessarily entangled with the restoration of pre-stroke features of motor-related cortical activity. As mentioned in our previous works (Cecchini et al., 2021; Conti et al., 2021b), we believe that the rehabilitation paradigm can promote different strategies in order to overcome the structural and functional alterations induced by the stroke damage.

In this work, we wanted to test the idea of whether neural activity in response to a motor output can be used to assess the effect of different therapeutic interventions following the induction of a focal cortical stroke. To our knowledge, this is the first time that such an approach, which is based on single-trial classification methods, has been attempted for wide-field calcium imaging data. Although the results obtained are promising, future work is needed to validate this approach using different therapies and compare the results to other measures that predict functional outcome. Moreover, even though we developed our method with calcium imaging data, which are not available in humans, the classification method proposed in this manuscript can be implemented using other neural signals such as EEG, fMRI, or FNIRs for better translational implementation.

## Data Availability Statement

The raw data supporting the conclusions of this article will be made available by the authors upon reasonable request.

## Ethics Statement

The animal study was reviewed and approved by the Italian Minister of Health, authorization n.183/2016-PR.

## Author Contributions

AS, AA, and FP conceived the study. EC collected the data. AS analyzed the data and generated the figures. AS and EC wrote the first draft of the manuscript. FP provided funding for the study. All authors contributed to manuscript revision, read, and approved the submitted version.

## Conflict of Interest

The authors declare that the research was conducted in the absence of any commercial or financial relationships that could be construed as a potential conflict of interest.

## Publisher’s Note

All claims expressed in this article are solely those of the authors and do not necessarily represent those of their affiliated organizations, or those of the publisher, the editors and the reviewers. Any product that may be evaluated in this article, or claim that may be made by its manufacturer, is not guaranteed or endorsed by the publisher.

## Supplementary Material

Supplementary Figure 1 Force and calcium response to the reward pull are similar across weeks for the rehab subjects. **(A)** Box plots of the force peak for the longitudinal data of the rehabilitated group. **(B)** Box plots of the fluorescence peak for the longitudinal data of the rehabilitated group. See Figure 2 legend for the description of the plots.

Supplementary Figure 2 Peak detection accuracy of the reward pull is not affected by the experimental group for a loso classification scheme. **(A)** Box plots of the peak accuracy for the three experimental groups. **(B)** Box plots of the peak accuracy for the longitudinal data of the rehabilitated group. See Figure 2 legend for the description of the plots.
