# Supplementary figures and images for: Tracking the Effect of Therapy With Single-Trial Based Classification After Stroke
Source: Front Syst Neurosci. 2022 May 4;16:840922. doi: 10.3389/fnsys.2022.840922 (PMC9114305; doi:10.3389/fnsys.2022.840922)

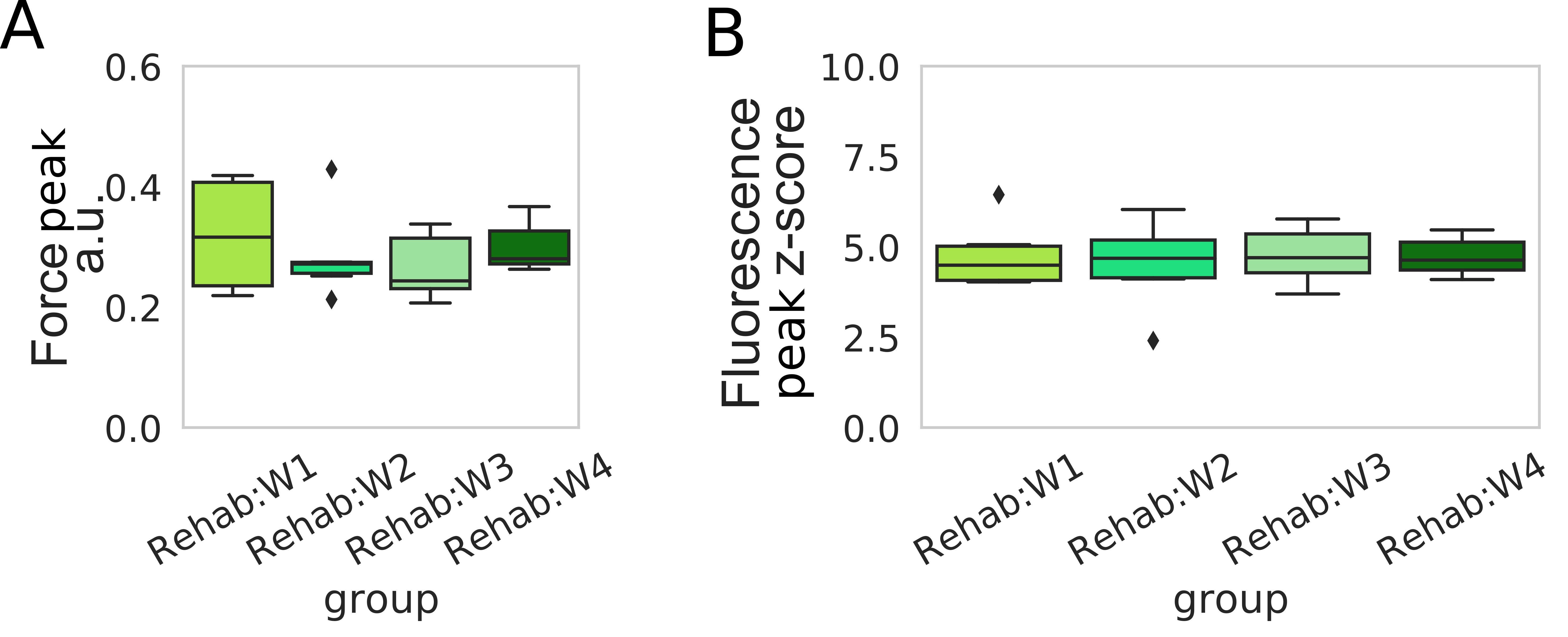

Supplement: Supplementary Figure 1 — Force and calcium response to the reward pull are similar across weeks for the rehab subjects. (A) Box plots of the force peak for the longitudinal data of the rehabilitated group. (B) Box plots of the fluorescence peak for the longitudinal data of the rehabilitated group. See Figure 2 legend for the description of the plots. [file Image_1.JPEG]

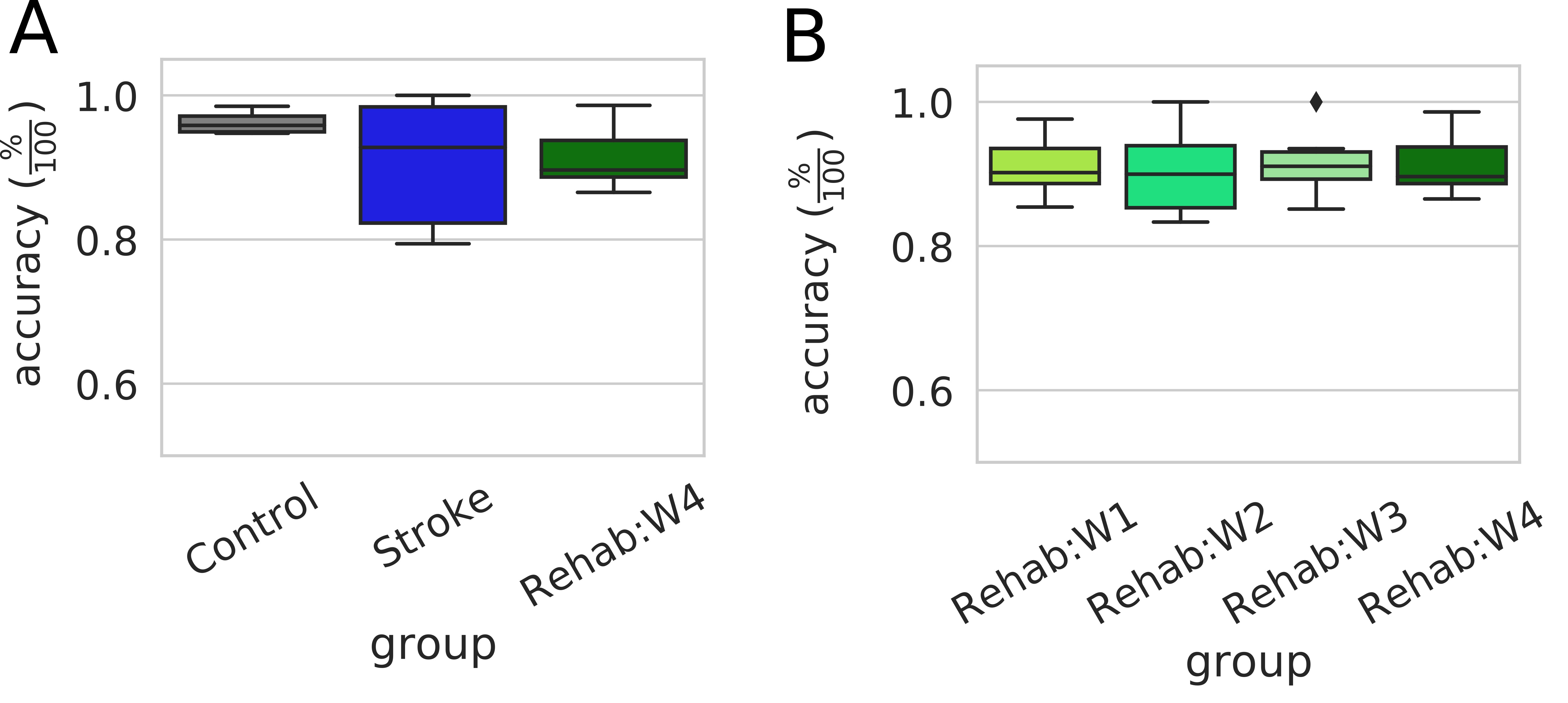

Supplement: Supplementary Figure 2 — Peak detection accuracy of the reward pull is not affected by the experimental group for a loso classification scheme. (A) Box plots of the peak accuracy for the three experimental groups. (B) Box plots of the peak accuracy for the longitudinal data of the rehabilitated group. See Figure 2 legend for the description of the plots. [file Image_2.JPEG]
